# Augmented Placental Protein 13 in Placental-Associated Extracellular Vesicles in Term and Preterm Preeclampsia Is Further Elevated by Corticosteroids

**DOI:** 10.3390/ijms241512051

**Published:** 2023-07-27

**Authors:** Marina Marks Kazatsker, Adi Sharabi-Nov, Hamutal Meiri, Rami Sammour, Marei Sammar

**Affiliations:** 1 Maternal and Fetal Medicine Unit, Department of Obstetrics and Gynecology, Bnai-Zion University Medical Center, Haifa 3498838, Israel; mkazatsker@gmail.com (M.M.K.); rsammour2002@gmail.com (R.S.); 2 Department of Statistics, Tel Hai Academic College, Tel Hai 122103, Israel; adi_nov@hotmail.com; 3 Department of Statistics, Ziv Medical Center, Safed 131000, Israel; 4 Hy-Laboratories Ltd., Rehovot 7670606, Israel; hamutal62@hotmail.com; 5 TeleMarpe Ltd., 21 Beit El St., Tel Aviv 6908742, Israel; 6 Prof. Ephraim Katzir Department of Biotechnology Engineering, Braude Academic College of Engineering, 51 Snunit St., Karmiel 2161002, Israel

**Keywords:** extracellular vesicles, placental protein 13 (PP13), galectin 13, preeclampsia (PE), preterm delivery (PTD), size exclusion chromatography (SEC), corticosteroids

## Abstract

Placental protein 13 (PP13) is a regulatory protein involved in remodeling the vascular system of the pregnancy and extending the immune tolerance of the mother to the growing fetus. PP13 is localized on the surface of the syncytiotrophoblast. An ex vivo placental model shows that the PP13 is released via placental-associated extracellular vesicles (PEVs) to the maternal uterine vein. This exploratory study aimed to determine PEV-associated PP13 in the maternal circulation as compared to the known soluble fraction since each has a specific communication pathway. Patients admitted to Bnai Zion Medical Center for delivery were recruited, and included 19 preeclampsia (PE) patients (7 preterm PE gestational age < 37 weeks’ gestation), 16 preterm delivery (PTD, delivery at GA < 37 weeks’ gestation), and 15 matched term delivery controls. Treatment by corticosteroids (Celestone), which is often given to patients with suspected preterm PE and PTD, was recorded. The PEV proteome was purified from the patients’ plasma by size exclusion chromatography (SEC) to separate the soluble and PEV-associated PP13. The total level of PP13 (soluble and PEV-associated) was determined using mild detergent that depleted the PEV proteome. PP13 fractions were determined by ELISA with PP13 specific antibodies. ELISA with alkaline phosphatase (PLAP)- and cluster differentiation 63 (CD63)-specific antibodies served to verify the placental origin of the PEVs. SPSS was used for statistical analysis. The patients’ medical, pregnancy, and delivery records in all groups were similar except, as expected, that a larger number of PE and PTD patients had smaller babies who were delivered earlier, and the PE patients had hypertension and proteinuria. The SEC analysis detected the presence of PP13 in the cargo of the PEVs and on their surface, in addition to the known soluble fraction. The median soluble PP13 was not significantly different across the PE, PTD, and term delivery control groups. However, after depleting the PEV of their proteome, the total PP13 (soluble and PEV-associated) was augmented in the cases of preterm PE, reaching 2153 pg/mL [IQR 1866–2838] but not in cases of PTD reaching 1576 pg/mL [1011–2014] or term delivery groups reaching 964 pg/mL [875–1636]), *p* < 0.01. On the surface of the circulating PEV from PTD patients, there was a decrease in PP13. Corticosteroid treatment was accompanied by a massive depletion of PP13 from the PEV, especially in preterm PE patients. This exploratory study is, thus, the first to determine PEV-associated PP13 in maternal circulation, providing a quantitative determination of the soluble and the PEV-associated fractions, and it shows that the latter is the larger. We found an increase in the amount of PP13 carried via the PEV-associated pathway in PE and PTD patients compared to term delivery cases, which was further augmented when the patients were treated with corticosteroids, especially in preterm PE. The signal conveyed by this novel communication pathway warrants further research to investigate these two differential pathways for the liberation of PP13.

## 1. Introduction

The interphase between the placenta and the maternal circulation has been extensively investigated in normal pregnancy and in pregnancy complications to obtain greater insights into the processes of placentation, fetal growth, maternal body adaptation to the pregnancy, and signal transmission between the pregnancy and the maternal organs [1,2]. Placental-specific galectins [1,3] and galectin 13, or placental protein 13 (PP13) in particular, are major regulatory proteins released by the placenta that communicate signals to the maternal organs about the well-being of the pregnancy. PP13 is specifically expressed by the syncytiotrophoblast and is first detected at GA 5–6 in the soluble maternal circulation. It is then gradually increased during gestation along with the increase in placental size (3–5). Studies have suggested that in preeclampsia (PE), and mainly in preterm PE requiring delivery before gestational week 37 (GA < 37 weeks’ gestation), there is a lower level of maternal blood PP13. Accordingly, the determination of PP13 in the first trimester was used as a marker for predicting the subsequent development of PE later in pregnancy. Near delivery with PE, there is a shedding of PP13 from the syncytiotrophoblast and the maternal blood circulation shows an increase in the level of PP13, at least in some studies [3,4,5].

Animal studies have found that PP13 expands the uterine and vascular arteries and veins, thus, priming the pregnancy for the required increase in the supply of oxygen and nutrients to the pregnancy [4,5]. PP13 has also been shown to be responsible for the mother’s immune system tolerance to the growing fetus [3,4]. Hence, PP13 is likely to play an important role in the fetal–maternal interactions during the course of normal and complicated pregnancies [3,5,6,7].

In the last ten years, studies have shown that extracellular vesicles (EVs) constitute the main communication vehicles between activated cell types, hormonal glands, tumors, fetal and placental cells, and between the immune system and remote organs [8,9]. These nanometer-sized membrane vesicles (exosomes and microvesicles) are released by the surface of certain cells and organs to convey signals (including possible complications at their site of origin) to remote organs. Their small size enables them to pass through small capillaries to enter the circulation, thus, transporting their cargo (RNA, proteins) and surface markers from their place of origin to remote organs. The study of EVs has, thus, contributed to major advances in the clinical management of diseases such as cancer, Alzheimer’s, COVID-19 vaccines, and central nervous system diseases. Advanced purification and subsequent size and surface characterization of EVs have led to the development of the current nomenclature of their size and surface shape [7,8,9].

This exploratory study focused on the evaluation of the communication pathway of the release of placental EVs (PEVs) that carry regulatory proteins, such as PP13, into the maternal circulation. Unlike our previous study, where we determined PP13 levels on the surface and in the internal cargo of PEVs collected after the ex vivo perfusion of the isolated placenta [6], here we focused on PEVs in the maternal circulation [7,8,9]. We compared the PP13 of the PEV in PE and preterm delivery (PTD) to term delivery.

PE is a major hypertensive disorder of pregnancy (affecting 2–8% of all pregnancies) [10,11]. Advances in the past decade have enabled better prediction of PE, especially preterm PE (PE requiring delivery before 37 week gestation) by first-trimester biomarkers and its prevention by low-dose aspirin [12,13]. Recent studies described the use of pro- and anti-angiogenic factors in identifying women at risk for developing PE in the third trimester [14]. Currently, most biomarkers of these complications are mainly determined using serum, whereas a plasma evaluation is less often conducted.

The current study focused on exploring the PEV-associated PP13, and determining changes in its maternal circulation level in PE, thus, providing an innovative diagnostic approach to the prediction of PE. In addition, we also evaluated the PEV-associated PP13 in cases of preterm delivery (PTD) [15,16], as a control for the preterm PE cases.

When we collected the data, we entered information on treatment with corticosteroids routinely used in the clinical management of PTD and preterm PE. The impact of corticosteroids was previously shown to affect the level of various regulatory proteins. Corticosteroids are mainly used to facilitate the maturation of the fetal respiratory system when preterm birth is suspected. There are reports that indicate various side effects following the use of corticosteroids [17,18,19,20,21,22]. We have previously reported that following corticosteroids’ treatment, there is a transient surge of maternal blood PP13 at different GAs, but the topic was not systematically followed [23,24].

Overall, this study had three goals: (1) identify the relative fractions of soluble and PEV-associated PP13 in the maternal circulation; (2) analyze the differential changes of the above in PE and PTD compared to term delivery controls; and (3) estimate the impact of corticosteroids on the levels of PP13 in each fraction.

## 2. Results

### 2.1. Cohort Characterization

**Figure 1:**
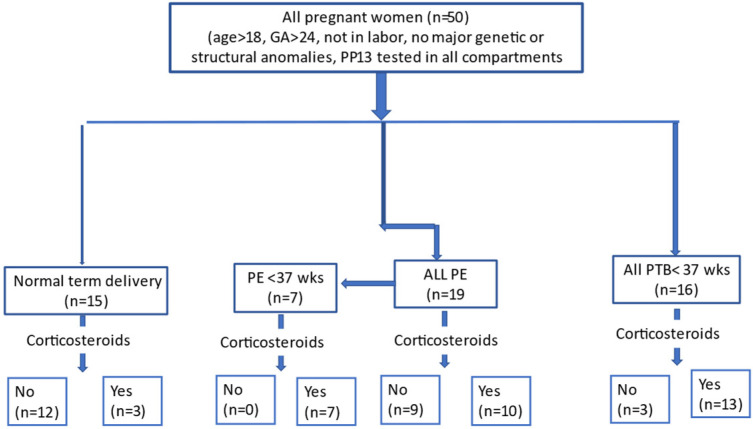
Study flow chart. We enrolled 50 patients—19 cases of PE, of whom 7 delivered at GA < 37 weeks’ gestation due to PE severity (PE < 37 wks), 16 PTD delivered at GA < 37 weeks’ gestation (PTD < 37 wks) unrelated to PE or other iatrogenic causes, and 15 term delivery controls. The number of women treated and untreated with corticosteroids (+/−) is shown.

We enrolled pregnant women attending the delivery clinic at Bnai Zion Medical Center (BZMC) with suspected PE and PTD compared to term delivery controls. The cohort included 19 PE cases (7 cases of preterm PE (delivery at GA < 37 weeks’ gestation), of whom 3 were delivered at GA 34 and below), and 16 cases of preterm delivery (all delivered at GA < 37 weeks’ gestation) of whom 3 were delivered before 34 weeks’ gestation compared to 15 cases of term delivery controls (Figure 1).

As shown in Table 1, the patients in the PE and PTD groups have a higher frequency of conception by in vitro fertilization (IVF), they deliver earlier, their babies are smaller, and a larger proportion of these newborns remain in the newborn intensive care unit (NICU) for at least one week. As expected, patients in the PE group have hypertension and proteinuria, but there are no other significant differences between the groups.

### 2.2. Quantitative ELISA Analysis of PP13 in Different Maternal Blood Fractions

In this exploratory study, we used size exclusion chromatography (SEC) to separate the soluble and PEV-associated PP13. The placental origin of the PEV was verified using the specific placental marker placental-associated alkaline phosphatase (PLAP). The identity of the EVs was further verified by cluster differentiation 63 (CD63). Mild detergent was used to deplete the PEVs from their PP13 cargo that was released to the plasma, thus, creating the total PP13 (PEVs associated and soluble combined). The PP13 on the surface of the PEVs was determined using small columns and SEC without detergent, a procedure that kept the PEVs intact to determine the surface PP13. We used ELISA to quantify the level of PP13 in each fraction (Supplementary Figure S1).

**Figure 2:**
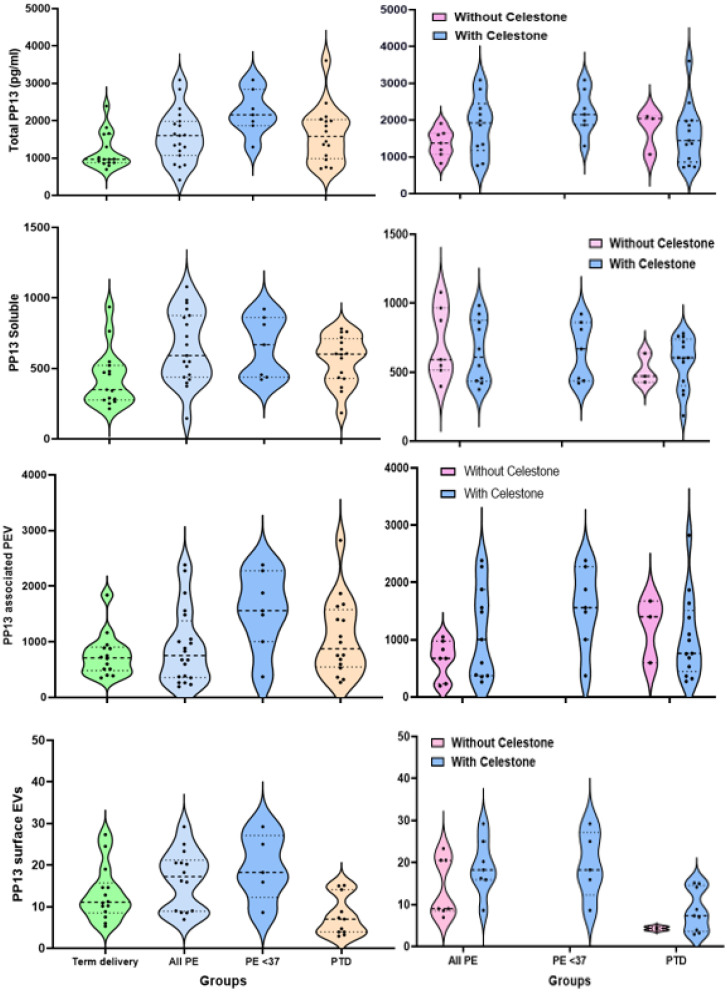
Violin plot of PP13 levels as a function of the different fractions. The four panels on the right side are the violin plots of the level of PP13 in the total fraction (top), the solubilized fraction (below), the PEV-associated fraction (below), while the very bottom plot is the PP13 of the surface of PEV. The four panels on the left side are the violin plots of the same PP13 fractions for the groups of patients treated with (pink) and without (blue) corticosteroids. In each violin plot, the horizontal dashed line represents the lower quartile, the median, and the third quartile. Dots along the violin show different patients’ values within the violin. The longitudinal lines on both violin apexes represent the minimum and the maximum values. PE—preeclampsia, PTD—preterm delivery, PE < 37—preterm PE, PEV—placental-associated extracellular vesicles.

As shown in Figure 2 and Table 2, the main finding is the significant increase in the total PP13 level compared to the soluble level. In the term delivery controls, the level of total PP13 is 964 pg/mL [IQR 875–1636], which is 2.5 times higher than the level of soluble PP13 fraction. In the group of All PE, the level of total PP13 is 1598 pg/mL [1070–1981], and it further augments to 2153 pg/mL [1866–2938, *p* < 0.01] in the preterm PE cases. The latter is higher than the total PP13 level in the PTD group, which is 1576 pg/mL [1011–2014].

Since the soluble levels of preterm PE cases compared to the PTD and term deliver control cases are not significantly different, the increase in the level of total PP13 in the preterm PE group is attributed to the increase in the level of PP13 in the PEV-associated PP13 fraction, which is what the PP13-associated fraction level shows (Table 3).

In the three cases of early PE (cases requiring delivery at GA < 34 weeks’ gestation, not shown), the level increases from 455 pg/mL [421–861] in the soluble fraction to 1981 pg/mL [1866–2838] in the total PP13 fraction. The increase in the total PP13 in the early PTD (cases delivered at GA < 34 weeks’ gestation) is only twice the soluble level. However, neither is significant (Table 2).

The results show that the cargo of PP13 in the PEV-associated PP13 is 699 pg/mL [IQR 511–891] in the term delivery controls compared to 830 pg/mL [355–1485] in all cases of PE, and 877 pg/mL [564–1519] in the PTD group (Table 2). In preterm PE, the PRV-associated cargo is the highest compared to the term delivery and the PTD groups. Accordingly, the PEV-associated pathway appears to be a major route for PP13 liberation to the maternal circulation in PE (Table 2). The PP13 level on the PEV surface is higher in PE, especially in preterm PE, whereas in the PTD group, the level decreases (Table 2, Figure 2).

### 2.3. The Impact of Corticosteroids

A decade ago, we found a temporary increase in the level of PP13 in patients treated with various corticosteroids [23,24]. Since cases of preterm PE and PTD were often treated with corticosteroids, we compared the relative fraction of PP13 levels between patients treated or untreated with corticosteroids (Table 3).

The results show a larger PP13 in PE patients treated with corticosteroids as compared to non-treated patients, which is true for all fractions (Table 3 and the right side of Figure 2). No comparison was available for preterm PE since they were all treated. In PTD, the surface of the PEV has higher PP13 and the soluble fraction appears higher but there is no significant difference in the total PP13 or the PEV-associated PP13 (Table 3, Figure 2, right side for each compartment).

## 3. Discussion

This exploratory study is the first to quantify the levels of PEV-associated PP13 in the maternal circulation in term delivery controls, in PE, and in PTD. This pathway is generated by the syncytiotrophoblast that sheds PEVs into the maternal circulation in addition to the previously reported pathway of soluble PP13 [3,25]. Previous studies have focused on the soluble PP13, and although the majority of these studies reported consistent results, the use of different analyzers and antibodies, and different methods of blood processing resulted in inconsistencies as to the usefulness of PP13 as a PE marker [3,25]. Here, it is found that the fraction of PEV-associated PP13 is the major pathway that carries the largest amount of PP13 from the placental origin into the maternal circulation. The amount carried by this pathway is higher in PE, especially in preterm PE cases, compared to the term or the preterm delivery cases.

While SEC is an analytical methodology that is not widely used in clinical labs, the same results were obtained by blood treatment with a mild detergent to generate the total PP13. The latter can easily be used in clinical labs. Hence, this study brings the PP13 marker back into the arena of predicting PE, with a verified overview of its actual presence in the maternal circulation, and a justified way to analyze it properly. This is important since PP13 is shown to be important for rendering the mother immune-suppressive to the growing fetus [25,26]. It is also important given there are studies that show how PP13 primes the uterine arteries and veins to increase the delivery of oxygen and nutrients to the placenta and CO_2_ and metabolite removal [27,28].

In a previous study, we used isolated placentae tested ex vivo and determined the level of PP13 in exosomes and microvesicles that were purified from the uterine vein after perfusing [6]. In that model, the levels of PP13 were normalized to the protein level and the data indicated that for a given amount of protein, the level of PP13 was lower on the surface and in the cargo of both the exosomes and microvesicles. Here, we show that near delivery, there is an increase in PEV-associated PP13 in PE cases, and primarily in preterm PE, compared to term delivery controls. This discrepancy can be resolved by the previously reported higher number of PEVs conveyed to the maternal circulation in PE versus term delivery controls [29,30]. Thus, it appears that while each individual PEV may carry less PP13, there is an overall increase due to the larger number of PEVs that are delivered into the maternal circulation.

At the research level, we are now developing a method for mounting the PEVs from the maternal circulation onto a glass surface of 96 well microplates and developing multiplex PEV arrays to fluorescently determine their amount and distribution by visualizing immune-labeled complexes and optical nanoscopy. The aim is to generate a novel analyzer of the risk of developing PE, which will be based on the PEV pathway between the placenta and the maternal circulation.

In the last ten years, EVs were shown to communicate signals carried internally or on their surface between remote organs in many differentiation and pathological conditions [29,30,31,32,33]. Here, we provide additional evidence that a specific type of EV, the PEVs, which are delivered from the placenta into the maternal circulation, should be further analyzed not only for PP13 but also for their RNA content [29] and other proteins such as placental growth factors (PlGF) and soluble FMS-like protein kinase-1 (sFlt-1), which are widely used in the prediction of PE near delivery according to their negative predictive values [14,34].

Corticosteroids are given to women attending a delivery admission clinic with suspected PTD or preterm PE. They are used to facilitate fetal lung maturation [17,18,19,20,21,22,23]. Here, we find that in PE, they augment the maternal blood levels of PP13. This may be linked to the cytokine storm or to the changes in the levels of TNF-alpha in PE [34,35,36], which are linked to the loss of immune tolerance in PE.

Limitations—This is an exploratory study, and we had no prior estimates of the anticipated level of PEV-associated PP13. We aimed for approximately the same number of patients in each group, which resulted in the fact that the study was underpowered. Although significant statistical differences were found, the small size of the groups may reflect an over-impact of certain individual patients. Thus, larger cohorts are needed to validate our findings in prospective studies.

Another limitation stems from the use of the mini-column for PEV purification, which is considered to be more analytical and not fully quantitative, since only a small amount of plasma could be loaded onto the mini-SEC columns. Subsequent studies are, thus, required to fully explore this more hidden reservoir of the PP13 on the surface of PEVs.

## 4. Materials and Methods

### 4.1. Sample and Patients

This study is exploratory. We had no previous information on the amount of PP13 in the PEV fraction, and, thus, aimed to have approximately the same number of patients in each group. Pregnant women attending the delivery clinic of Bnai Zion Medical Center (BZMC) in Haifa were invited to take part between August 2020 and May 2022. The term delivery controls were enrolled on the same or the next day as the study groups (to avoid bias). The enrollment criteria were GA of 24 weeks and above, and not being in labor when enrolled in the study. Other inclusion criteria were a maternal age of 18 years and above, viable singleton pregnancies without major fetal structural and genetic abnormalities, and the patients’ agreement to undergo all test procedures and deliver at the medical center. The exclusion criteria were multiple pregnancies, fetal abnormalities, preexisting renal, hematological, autoimmune, or severe cardiovascular conditions, or the inability to sign the informed consent due to mental disabilities. GA was determined from the last menstrual period and was verified by evaluating the records of the routine first-trimester ultrasound of the fetal crown–rump length [37].

The study was approved by the Ethics Committee of BZMC (approval # BZMC-0107-19), and informed written consent was obtained from all participants. Demographics, medical, and pregnancy history, and delivery records were extracted from the hospital’s electronic medical records. These included the drugs taken during pregnancy such as low-dose aspirin, vaginal progesterone, tocolysis, and corticosteroids.

Blood pressure was measured at the time of enrollment with a Welch Allyn Nonin SPo2 device. These devices are calibrated regularly as per protocol. Measurements were made according to the guidelines of the Fetal Medicine Foundation (FMF), which advises measuring the diastolic and systolic blood pressure twice, 20 min apart, and calculating the diastolic, systolic, and mean arterial blood pressure [38].

The pregnancy complications are outlined below.

Preeclampsia (PE)—data on pregnancy outcomes were obtained from hospital records. PE was diagnosed according to the guidelines of the International Society for the Study of Hypertension in Pregnancy (ISSHP). According to this definition, the diagnosis of PE requires the presence of new-onset hypertension (systolic blood pressure ≥ 140 mmHg or diastolic blood pressure ≥ 90 mmHg) at GA ≥ 20 weeks’ gestation, or chronic hypertension and either proteinuria (≥300 mg/24 h or protein-to-creatinine ratio ≥ 30 mg/mmol or ≥2+ on dipstick testing), or evidence of renal dysfunction (serum creatinine > 97 μmol/L), hepatic dysfunction (transaminases ≥ 65 IU/L), or hematological dysfunction (platelet count < 100,000/μL). Outcome measures were all PE, preterm, and early PE, with delivery at any gestation, or at <37, and <34 weeks’ gestation [11,12,13].

Preterm delivery (PTD) was defined as delivery before 37 weeks’ gestation [14,16] that was not related to fetal growth restriction or PE, chorioamnionitis, placenta abruption, placenta previa, or placenta accreta.

### 4.2. Blood Drawing and Processing

At the time of enrollment, 10 mL of whole blood was drawn into K_2_EDTA tubes (BD, Heidelberg, Germany), turned upside down several times to assure a good mixture of the blood with the solution, then centrifuged at 1500× *g* for 10 min at RT. The clear plasma was aspired and stored in 0.5 mL cryovials at −80 °C until use, with labels listing the patient’s code and the date of sample collection.

### 4.3. The Different Fractions of PP13

**Figure 3:**
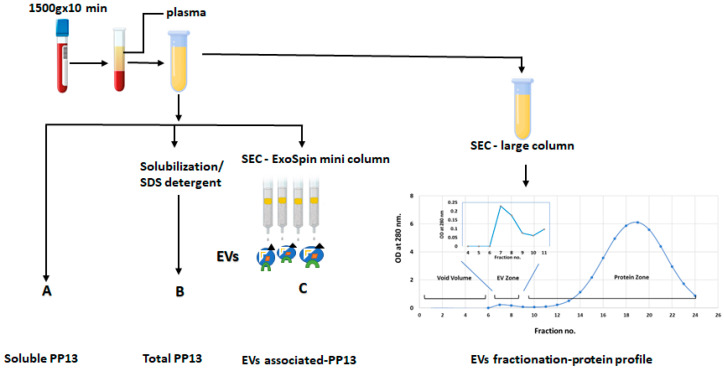
Sub-fractionation of maternal plasma. Starting from whole blood, the plasma was collected by EDTA-containing tubes. A–C: PP13 level was determined by ELISA in the soluble compartment (A). The total PP13 was determined by ELISA after treating the plasma with mild detergent to deplete PP13 from the PEV (B). Exo Spin mini-size exclusion chromatography (SEC) columns were used to exclusively isolate the PEV and determine PP13 on their surface (C). Note that in C, the level was limited by column capacity. To the right side, we describe the entire process of SEC while the actual SEC profile is shown in the Supplementary Figure S1. The schematic profile at the lower right side below the SEC large column tube indicates the zone of fractions 6–8 of the SEC includes the PEVs while the zone of fractions 12–24 include the soluble proteins (further details are demonstrated in Supplementary Figure S1).

We developed a step-wise method to determine PP13 in the different fractions of maternal blood (Figure 3). Soluble PP13 was measured directly. PEV-associated PP13 was obtained by SEC. Treatment with mild detergent yielded a total PP13. PP13 of the surface PEVs was determined on mini-SEC columns (Figure 3).

### 4.4. Size Exclusion Chromatography

The extracellular vesicles (EVs) from maternal plasma were isolated by size exclusion chromatography (SEC) according to the manufacturer’s instructions (Izon Science, Lyon, France). Plasma samples were thawed on ice and centrifuged at 2000× *g* for 10 min to remove aggregates generated during the freeze/thaw cycle. The supernatant was filtered using a 0.22 µm constant well filtration system after centrifugation. Then, 0.5 mL of plasma was loaded on the top of the pre-equilibrated EV column (Izon Science). The column was washed with 3 mL PBS to collect 0.5 mL fractions of the void volume. Filtered PBS was added into the column and 24 fractions of 0.5 mL each were collected as eluents. The protein content was monitored in each fraction by measuring the optical density OD at 280 nm. Protein concentration was determined by BCA assay (Thermo-Fisher, Waltham, MA, USA). All fractions were stored at −80 °C for downstream analyses.

### 4.5. Isolation and Characterization of EVs

The isolation of EVs from maternal blood was performed by Exo-spin™ 96 according to the manufacturer’s instructions (Cell Guidance Systems, Cambridge, UK). Briefly, the plasma was centrifuged at 16,000× *g* for 30 min to remove any remaining cell debris and large aggregates. Aliquots of 100 μL of the centrifuged plasma were loaded on each pre-equilibrated column and allowed to enter the column. The flow-through was then collected into the waste plate and discarded. PBS (180 μL) was applied to each column and the exosomes were eluted into the collection plate. The protein content in the eluate was monitored by measurement of the OD at 280 nm. Samples were stored at −70 °C until use.

### 4.6. Solubilization of PEVs

To quantify the total PP13 in the plasma (soluble and PEV-associated PP13), the plasma was treated with 0.1% sodium dodecyl sulphate-SDS/PBS as a detergent for 30 min on ice to solubilize the EVs and release the PP13 content in the EV compartments (inside and outside) [39]. Total PP13 was determined in the solubilized proteins by ELISA as described in Section 4.6 below. The profile of the SEC before and after detergent treatment is shown in Supplementary Figure S1.

### 4.7. Quantification of PP13 by ELISA

Determination of the total PP13, soluble PP13, and EV-associated PP13 concentrations was performed by a competitive enzyme-associated immunosorbent assay (ELISA) according to the manufacturer’s instructions (Cusabio, Wuhan, China; cat. #: CSB-E12733h). All the samples were analyzed in duplicate. Briefly, the wells of the ELISA plates precoated by the manufacturer with anti-PP13 antibodies were incubated for 2 h, with the diluted samples together with PP13 conjugated to HRP to allow for the competition between PP13 in the plasma samples labeled PP13 to reach equilibrium. Access reagents were washed and 3,3′,5,5′ tetramethylbenzidine (TMB) chromogenic substrate was added. The reaction was stopped by 2 N HCL and the optical density of the colored developer was determined at 450 nm using an ELISA plate reader. PP13 concentration was calculated based on the standard curve generated in the same experiment.

Indirect ELISA was used to determine the PP13, PLAP, and CD63 of the SEC-eluted fractions [1,2,3,4,5,6,7,8,9,10,11,12,13,14,15,16,17,18,19,20,21,22,23,24]. For this assay, the SEC fractions were diluted 1:50 in a 50 mM carbonate buffer at pH 9.6 and were coated in duplicate on the wells of flat-bottom ELISA plates (Falcon, BD Heidelberg, Germany). The SEC diluted fractions were left for overnight incubation at 4 °C followed by blocking of the nonspecific binding sites with 2% bovine serum albumin (BSA) in phosphate-buffered saline (PBS). The three pairs of SEC fractions were incubated with either anti-PP13 antibodies at 0.1 microgram /mL of monoclonal anti-PP13 (clones 534 and 215-28-3) [26,29,30,31,32,33,34,35,36,37,38,40,41,42,43,44,45,46], or anti-PLAP antibody (NDOGE2, a generous gift from Dr. Manu Vatish, Oxford University [6,9]) or the commercially available anti-CD63 (Thermos Fischer Scientific, Waltham, MA, USA) for 2 h at room temperature (RT). After washing off excess reagents, the plates were incubated for 1 h with goat anti-mouse IgG conjugated with horseradish peroxidase, HRP (Dianova, Königswinter, Germany) for 1 h at RT. Extensive washing with PBS containing 0.05% Tween was performed between steps. The reaction product was developed with TMB substrate (Thermo Fisher Scientific, Waltham, MA, USA), stopped with 2 N HCl, and the optical density was measured using a microplate spectrophotometer reader (BioTeck instruments Inc., Santa Clara, CA, USA) at 450 nm.

### 4.8. Statistics

The data were analyzed using the SPSS version 28 (IBM, Chicago, IL, USA). For descriptive statistics, the categorical variables are presented as frequencies (n) and percentages, whereas the continuous variables are presented as the median or medians and interquartile range [IQR]. For the inferential statistics, differences between groups for the continuous variables were examined using a Kruskal–Wallis or a Mann–Whitney non-parametric test. Relationships between groups and the categorical variables, and were calculated using chi-square tests or the Fisher exact test, depending on sample size.

A violin plot was used to describe the medians, quartiles, and the upper and lower range. The advantage of the violin plot over the box plot is that it not only shows the medians, quartiles, and ranges but also depicts the cases included in each quartile, which is more informative than box plots where the box size is fixed.

## 5. Conclusions

**Figure 4:**
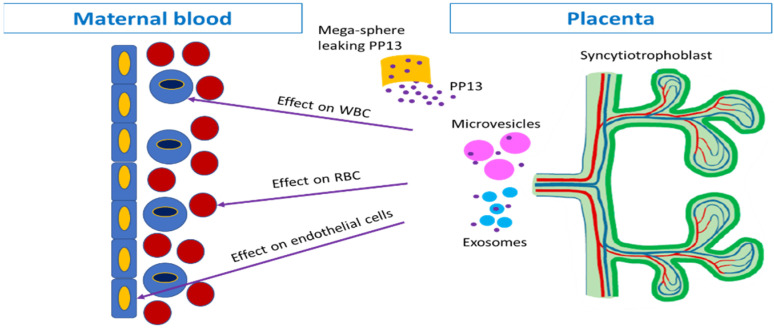
PP13 pathways to the maternal circulation. The placenta syncytiotrophoblast (green to the right) releases PP13 through the uterine vein into the maternal circulation (the blue blood vessel to the left). The PEVs serve as the communication pathway. The microvesicles (pink) and the exosomes (turquoise) carry PP13 (purple) on their surface and inside. There is also a leakage of soluble PP13 (purple) either into the intracellular space or through necrotic vesicles (orange) that are too big to pass through the capillaries, thus, leaking their content into the soluble fraction of the maternal circulation. PP13 is, thus, liberated either as a soluble protein or in association with the PEVs. Clinical pathologies affect PP13 mainly via the PEV pathway. When reaching the maternal circulation, PP13 binds to the ABO antigen on the red blood cells (RBC, [47]), the white blood cells (WBC [3,26]), or the endothelial layer [27,28,48], Sammar et al. Elements of this figure were modified from our former review Sammar et al. [7] *Int. J. Mol. Sci.* **2019**, *20*, 3192. https://doi.org/10.3390/ijms20133192.

This exploratory study was designed to quantify the pathway of PEVs carrying PP13 on their surface and in their inside cargo into the maternal circulation (Figure 4). PP13 carried via this pathway in PE and PTD patients is higher than in the term delivery controls, especially among preterm PE cases. When the patients were treated with corticosteroids, a further increase in PP13 liberation was found. These results illustrate that the PEVs create an important communication pathway between the placenta and maternal circulation, and this finding warrants further research.

## Figures and Tables

**Table 1:** Characterization of the study population—pregnancy and delivery features.

Parameter	Term Control Delivery at GA> 37 wks (n = 15)	All PE (n = 19)	Preterm PE Delivery < 37 wks (n = 7)	PTD Delivery < 37 wks (n = 16)	*p*
Enrolment					
Maternal age (years)	30.0 [27.0–33.0]	32.0 [30.0–36.0]	32.0 [30.0–36.0]	30.5 [28.0–35.0]	0.244
BMI (kg/h^2^)	25.0 [22.2–32.3]	32.0 [26.0–36.0]	27.0 [22.0–34.0]	27.0 [26.0–29.0]	0.449
BMI > 30 n (%)	4 (33.3)	11 (57.9)	2 (28.6)	2 (13.3)	0.189
Smoker n (%)	4 (26.7)	10 (52.6)	3 (42.9)	9 (56.3)	0.592
Previous PE n (%)	0 (0)	5 (26.3)	4 (57.1)	5 (31.3)	0.151
Null parity n (%)	2 (13.3)	0 (0)	0 (0)	0 (0)	0.247
Conception, IVF n (%)	0 (0)	1 (5.3)	0 (0)	0 (0)	0.798
Previous GDM n (%)	2 (13.3)	2 (10.5)	0 (0)	2 (12.5)	0.765
Chronic hypertension 0, (%)	0 (0)	3 (15.8)	1 (14.3)	0 (0)	0.301
Diabetes mellitus 0 (%)	2 (13.3)	1 (5.3)	0 (0)	0 (0)	0.503
Systolic BP (mm Hg)	109 ^b^ [105–119]	162 ^a^ [150–180]	180 ^a^ [133–195]	123 ^b^ [115–128]	**<0.001**
Diastolic BP (mm Hg)	71 ^b^ [69–75]	100 ^a^ [90–107]	107 ^a^ [82–110]	77 ^b^ [73–80]	<0.001
MAP (mm Hg)	84 ^b^ [81–90]	118 ^a^ [108–133]	133 ^a^ [99–136]	91 ^b^ [88–96]	<0.001
Creatinine (mg/dL)	0.50 ^b^ [0.4–0.6]	0.70 ^a^ [0.6–0.8]	0.8 ^a^ [0.6–0.8]	0.5 ^b^ [0.5–0.6]	0.004
Aspirin n (%)	0 (0)	3 (17.6)	2 (33.3)	1 (6.3)	0.020
Progesterone ^#^ n (%)	3 (20.0)	0 (0)	0 (0)	1 (6.3)	0.115
Delivery					
GA at delivery (wks)	39.1 ^a ^ [38.7–39.9]	37.1 ^b^ [35.0–37.4]	34.3 ^c ^ [32.9–35.6]	36.2 ^b ^ [34.7–36.6]	<0.001
Infant’s birthweight (gr)	3290 ^a^ [2840–3620]	2730 ^b^ [2180–3125]	2100 ^c^ [1830–2240]	2498 ^b^ [2235–3068]	<0.001
Infant gender (male) n (%)	10 (66.7)	10 (55.6)	5 (71.4)	16 (100)	0.036
NICU days	39.1 ^a ^ [38.7–39.9]	37.1 ^b^ [35.0–37.4]	34.3 ^c^ [32.9–35.6]	36.2 ^b ^ [34.7–36.6]	<0.001

Continuous variables are shown as medians and the interquartile range [IQR], and categorical variables are shown as frequencies—n, and percentages (%). ^#^ Progesterone was given for a short cervix. The letter letters “^a^” to “^c^” represent significant differences between the groups’ medians using the Kruskal–Wallis non-parametric test. The letter “^a^” is significantly higher, “^b^” is significantly lower than “^a^”, and “^c^” is lower than all. PE—preeclampsia, PTD—preterm delivery. BMI—body mass index, MAP—mean arterial blood pressure. IVF—in vitro fertilization, NICU—newborn intensive care unit, GA—gestational week, BP—blood pressure, GDM- gestational diabetes mellitous, NICU—newborn intensive care unit.

**Table 2:** Placenta protein 13 (PP13) levels according to groups and fractions.

	Groups	Term Control (Delivery > 37wks)	All Preeclampsia	Preterm PE (Delivery < 37 wks)	PTD (Delivery < 37 wks)
Fractions		(n = 15)	(n = 19)	(n = 7)	(n = 16)
PP13 soluble		349 [276–522]	591 [437–875]	668 [437–861]	602 [431–701]
Total PP13		964 [875–1636]	1598 [1070–1981]	2153 * [1886–2838]	1576 [1011–2014]
PP13-associated PEV		699 [511–891]	830 [355–1485]	1560 * [1004–2277]	877 [564–1519]
Surface PEVs PP13		11.1 [8.8–14.6]	17.2 [9.0–20.5]	18.2 * [15.9–25.0]	7.0 * [3.9–14.1]

Results are shown as medians and the interquartile range [IQR]. * Significantly different from the control group using a Mann–Whitney non-parametric test (*p* < 0.01). PE: preeclampsia, PTD: preterm delivery, PEV: placental-associated extracellular vesicles. PP13-associated PEV was obtained by subtracting the soluble PP13 from the total PP13. Surface PEV PP13 was determined on aliquots of mini-SEC columns.

**Table 3:** Placenta protein 13 (PP13) levels as a function of treatment with corticosteroids.

Group	Term Delivery (n = 15)		PE (n = 19)		Preterm PE (n = 7)		PTD (n = 16)		*p*	*p*

Treatment	Celestone		Celestone		Celestone		Celestone		Celestone	
	+	-	+	-	+	-	+	-	+	-
**Fraction**	**(n = 3)**	**(n = 12)**	**(n = 10)**	**(n = 9)**	**(n = 7)**	**(n = 0)**	**(n = 13)**	**(n = 3)**		
Total PP13	911 [875–1636]	967 [866–1475]	1923 [1293–2316]	1372 [1070–1640]	2153 [1866–2838]		1442 [655–1973]	2037 [1068–2102]	0.129	0.150
PP13 soluble	471 [276–522]	345 ^b^ [274–513]	608 [437–861]	591 ^a^ [514–966]	668 [437–861]		604 [435–720]	471 ^ab^ [427–636]	0.425	0.041
PP13-associated PEV	599 [390–1164]	711 [511–884]	1245 [372–1879]	676 [233–975]	1560 [1004–2277]		763 [531–1387]	1401 [597–1676]	0.365	0.318
Surface PEVs PP13	10.1 ^ab^ [5.3–27.3]	11.2 ^a^ [8.8–14.6]	18.2 ^a^ [15.9–25.0]	14.7 ^a^ [8.5–20.5]	18.2 ^a^ [15.9–25.0]		7.3 ^b^ [3.9–14.1]	4.3 ^b^ [3.9–4.7]	0.017	0.075

Results are shown as medians and the interquartile range [IQR] using the Kruskal–Wallis non-parametric test. The letter “^a^” stands for significantly higher, “^ab^” is also significantly higher but lower than the values marked “^a^”, “^b^” is significantly lower than any. PE: preeclampsia, PTD: preterm delivery. Enzyme-linked immunosorbent assay (ELISA) was used for the quantification of PP13 in each fraction. Total PP13 was obtained after treatment with mild detergent. Soluble PP13 was determined directly. The PEV-associated PP13 is the total PP13 minus the soluble PP13. The surface PEVs PP13 was determined on aliquots of mini-SEC columns.

## Data Availability

Not applicable.

## Supplementary Materials

The following supporting information can be downloaded at: https://www.mdpi.com/article/10.3390/ijms241512051/s1.

## Abbreviations

ACOG	American College of Obstetricians and Gynecologists
BMI	Body mass index
BP	Blood pressure
BZMC	Bnai Zion Medical Center
CD63	Cluster Differentiation 63
EVs	Extracellular Vesicles
ELISA	Enzyme-linked Immuno Sorbent Assay
FGR	Fetal Growth Restriction
GA	Gestational Age
GDM	Gestational Diabetes Mellitous
ISSHP	International Society for the Study of Hypertension Disorders in Pregnancy
ISUOG	International Society for Ultrasound in Obstetrics and Gynecology
IVF	In Vitro Fertilization
MAP	Mean arterial blood pressure
NICU	Newborn Intensive Care Unit
NPV	Negative Predictive value
PE	Preeclampsia
PEVs	Placental-associated Extracellular Vesicles
PlGF	Placental Growth Factor
PLAP	Placental-derived Alkaline Phosphatase.
PP13	Placental Protein 13
PTD	Preterm Delivery
sFlt-1	Soluble FMS (oncogene for Feline McDonough Sarcoma)-like tyrosine kinase 1
SEC	Size Exclusion Chromatography
