# Supplementary material for: Augmented Placental Protein 13 in Placental-Associated Extracellular Vesicles in Term and Preterm Preeclampsia Is Further Elevated by Corticosteroids
Source: Int J Mol Sci. 2023 Jul 27;24(15):12051. doi: 10.3390/ijms241512051 (PMC10419231; doi:10.3390/ijms241512051)
Supplement: Supplementary file 1 [file ijms-24-12051-s001.zip › ijms-2449099-supplementary.docx]

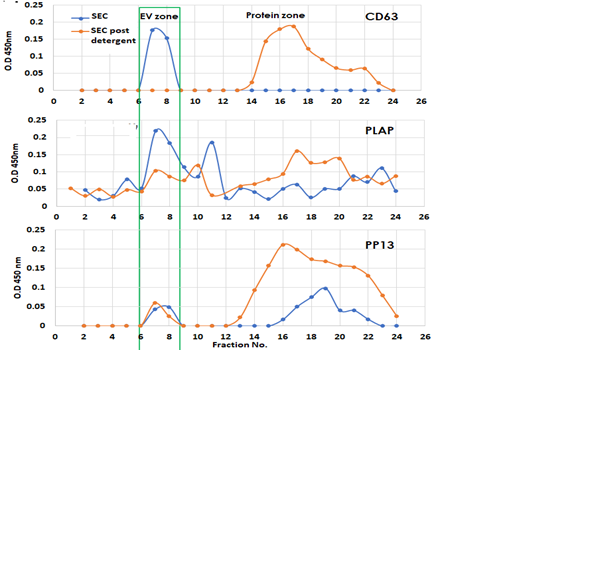


Supplementary Figure S1: Size exclusion chromatography (SEC) of placental derived EVs (PEVs). The SEC was carried out before and after SDS treatment and the content in each sub-fraction (fractions 6-10, EV zone) and (fractions 11-24, protein zone) was determined with specific markers by ELISA: PP13, CD63 (EV marker(, and PLAP )placental marker(. The profile of the fractionation is shown before (blue line) and after (orange line) treatment with detergent. The detergent treatment depletes the EVs from their proteome, and the profile revealed how each of these proteins is divided between the soluble and the PEV zones before and after the detergent treatment.
